# Modified Vertical Rectus Abdominis Musculocutaneous Flap for Limb Salvage Procedures in Proximal Lower Limb Musculoskeletal Sarcomas

**DOI:** 10.1155/2008/781408

**Published:** 2008-03-12

**Authors:** Haitham H. Khalil, Ahmed El-Ghoneimy, Yasser Farid, Walid Ebeid, Ahmed Afifi, Ahmed Elaffandi, Tarek Mahboub

**Affiliations:** ^1^ Department of General Surgery, King's College Hospital, Denmark Hill, London SE59RS, UK; ^2^Department of Plastic and Reconstructive Surgery, Cairo University Teaching Hospital, Cairo University, Kasr Al Ainy, Cairo 11559, Egypt; ^3^Center of Preservation and Musculoskeletal Tissue Transfer, Cairo University Teaching Hospital, Kasr Al Ainy, Cairo University, Cairo 11559, Egypt; ^4^Division of Plastic & Reconstructive Surgery, University of Pittsburgh Medical Center, Pittsburgh, PA 15213, USA; ^5^Department of Surgery, Royal Free Hospital, London NW3 2PN, UK

## Abstract

*Introduction and aim*. Management of complicated wounds after tumor extipiration of pelvic and proximal lower limb musculoskeletal sarcoma represents an essential component in the outcome of these patients. The authors present modified vertical rectus abdominis musculocutaneous (VRAM) flap techniques to reconstruct extensive defects after debridment of these complicated wounds. *Material and Methods*. Over a period of 4 years (2002–2005), 5 men and 2 women were managed. Median age was 21 years (range 15–49). The patients were managed for complicated lower trunk, groin, and upper thigh wounds after resection of three pelvic chondrosarcomas as well as two pelvic and two proximal femur osteosarcomas. The modifications included a VRAM flap with lateral and tongue-like extension design of the skin paddle (5 cases) or a delayed extended VRAM flap (2 cases). *Results*. All flaps showed complete survival and healing with no ischemic events providing stable coverage. All patients were ambulant with good limb functions in terms of walking and gait after adequate rehabilitation, 2 needed support with crutches. *Conclusion*. The modified VRAM flaps offer reliable reconstructive tools for coverage of complex groin and thigh defects by providing larger well-vascularized soft tissue with acceptable donor site.

## 1. INTRODUCTION

Limb salvage remains a major objective in contemporary management of bony and soft tissue sarcomas of the pelvis and lower extremities [1, 2]. Radical oncological salvage procedures performed in this area can develop significant wound complications that result from a high incidence of infection, dehiscence, or flap necrosis aggravated by the paucity of well-vascularized local soft tissue and systemically immune-compromised patients [3–6]. The reconstruction of extensive and complex wounds as a secondary event after tumor extipiration represents a challenging problem for both the orthopaedic and reconstructive surgeons [7]. This would thereby jeopardize the long-term wound healing with delay in the postoperative adjuvant chemotherapy and eventually the successful outcome. The authors present two modifications of the standard VRAM flap for coverage of extensive potentially infected defects after radical debridement of complicated groin and upper thigh wounds. The modifications included an inferiorly based VRAM flap with lateral and tongue-like extension design of the skin paddle or a delayed extended VRAM flap.

## 2. MATERIAL AND METHODS

Seven consecutive patients with complicated wounds secondary to resection of pelvic and proximal lower extremity musculoskeletal sarcoma were managed with modified VRAM flaps between 2002–2005. The median age of the patients in this series at the time of surgery was 21 years (range 15–49) and included 5 men and 2 women. The modifications included VRAM flap with lateral and tongue-like extension design of the skin paddle (5 cases) or a delayed extended VRAM flap (2 cases). All the patients have been treated with a multidisciplinary approach; the management of osteosarcoma patients (2 pelvic, 2 proximal femur) included a 4-month course of neoadjavant chemotherapy followed by resection of the primary tumor and postoperative 3-month course of chemotherapy; while chondrosarcoma patients (3 pelvic) were offered surgical resection only. The complications, which necessitated management by the plastic and reconstructive surgeons, included infection and wound dehiscence with ultimate exposure of metal prosthesis (4 patients) and necrosis of thigh flaps (3 patients). The median interval duration between the primary operation and the reconstructive procedure for the complicated wounds was 16 days (range: 13–36). All patients underwent thorough clinical assessment, adequate debridment and irrigation of the wounds, followed by immediate coverage of the resultant defects (median size 7 × 17 cm) using modified VRAM flaps.

## 3. DETAILS OF MODIFIED TECHNIQUES

### 3.1. Delayed extended VRAM technique (2 cases) (Figure 1)

This technique was used to cover exposed metal plates due to wound breakdown after osteosarcomas resection of proximal femur where length of the flap was predominantly required than its width. An ipsilateral parasternal skin paddle was designed 10 cm cephalic to the costal margin and 2 cm from the lateral sternal edge. The pinch test, originally described by Taylor et al. [8], was used to determine the maximum width of the skin paddle allowing primary closure of the donor site [5]. A complete semicircumferential incision (surgical delay) was done down to the pectoral fascia. The parasternal fasciocutaneous flap was then harvested starting 10 cm cephalic to the costal margin with caudal dissection in suprapectoral fascial plane. This was followed by transecting the costal origin of rectus muscle and ligation of the superior epigastric pedicle (vascular delay) leaving the parasternal extension flap continuous with the skin paddle of the standard VRAM. The flap was resutured and monitored for 2 weeks to identify the line of demarcation between augmented perfused and nonperfused skin. The second stage involved excision of the nonperfused skin (25–30%) of the parasternal extension flap, refreshing the skin flap incision from the delay procedure followed by harvesting VRAM flap utilizing the standard principles. Special attention was paid when incising the medial and lateral borders of the rectus muscle longitudinally along its entire length so as to leave a 1 cm of sheath along each border for closure. The inferior epigastric pedicle was skeletonised; thus providing the pivot point for rotation of the flap. The VRAM flap throughout its whole length was harvested in continuity with the augmented parasternal fasciocutaneous component leaving the posterior rectus sheath in situ providing an additional average length of 7 cm. Tension-free closure of the donor site was achieved by undermining the abdominal skin laterally beyond the margins of the rectus abdominis muscle in the fascial plane.

### 3.2. Laterally oriented VRAM with tongue-like skin extension technique (5 cases) (Figure 2)

This technique was used in cases after resection of chondrosarcomas and osteosarcomas of the pelvis, which necessitated the development of large subcutaneous thigh flap to allow adequate exposure and vascular exploration to achieve adequate oncological resection. Extensive full-thickness thigh flap necrosis occurred in these patients which necessitated adequate debridement and coverage of the resultant defect utilizing a contralateral, laterally oriented VRAM with a tongue-like skin extension. The contralateral muscle was selected due to the sacrifice of the ipsilateral inferior epigastric pedicle as part of the radicality of the primary tumor resection. The lateral orientation of the skin paddle was designed to be oblique (45°) to the longitudinal axis of the rectus muscle to provide an average extra length of 5 cm. The tongue-like extension of the skin paddle was designed throughout the whole length of the muscle tapering towards the pivot point of the flap at the entry of the inferior epigastric pedicle. The fasciomusculocutaneous component of the VRAM flap was harvested utilizing the standard technique with elevation of the laterally oriented part of the flap in the fascial plane. The origin and insertion of rectus muscle were transacted leaving the vascular pedicle as the sole tether point; this significantly increased the arc of rotation allowing more muscle mass to be transposed to the wound. Incising the skin bridge between the donor and recipient defect to lay open the tunnel connecting the recipient and the donor site followed this. The tongue-like skin extension provided the roof for opened tunnel after rotation of the flap to settle to the defect; allowing more room for the pedicle and providing coverage for the medial part of the defect.

In all patients, the donor site was closed primarily after reinforcement of the abdominal wall using an onlay prolene mesh, suction drains were used both at the recipient and donor sites. All patients were mobilized within 2 weeks postoperatively under supervision of the orthopaedic and physical therapy team. The mean duration to postoperative chemotherapy was 3 weeks. The mean follow-up period was 26 months at which patients were followed up with assessment of the oncological outcome, durability of soft tissue reconstruction, and finally assessing the functional outcome using Enneking system,

## 4. RESULTS

All flaps showed complete survival and healing with no ischemic events, hence providing stable coverage. Mild infection was observed in 1 patient; no haematomas were detected. All patients were ambulant with good limb functions in terms of walking and gait after adequate rehabilitation; 2 needed additional support with crutches. All patients showed good emotional acceptance; and no pain has been observed during ambulation in all patients. One patient developed incisional hernia at the donor site that required repair 1 year later; otherwise, no other donor site morbidity was noted. Another patient died of haematogenous metastatic spread after complete resection 15 months later. There was no hypertrophic scar formation or flap breakdown experienced on long-term follow-up in any of the patients.

## 5. DISCUSSION

Limb preservation has become a more realistic and necessary goal in the management of patients with musculoskeletal sarcoma of extremities [9]. Radiation and chemotherapy in addition to limb preservation through recent refinement in reconstructive surgery have improved the local, systemic control and the subsequent overall survival [10, 11]. The most common complications of limb salvage procedures for musculoskeletal sarcomas surgery have been problems with wound healing, flap necrosis, and wound infections [1, 6]. Infections associated with prosthetic replacement after tumor resection in these patients are common and serious complications as they are often subjected to extensive soft tissue dissection and long operating times while systemically immunocompromised [5, 6, 12].

The reconstructive options to provide coverage following debridment of these complicated wounds would be local, distant flaps, or free-tissue transfer. VRAM flaps have been used successfully in coverage of defects of the chest wall, groin, hip, perineal, vaginal, and gluteal regions with good functional outcomes [13–17]. The advantages of pedicled standard VRAM are providing ample skin, soft tissue bulk, simplicity of execution, low complication rate, and high success rate [18] in which a safe and fast forward flap procedure is the reconstructive goal especially in high-risk group. In this study, modifications have been applied to the standard VRAM flaps in an attempt to mobilize larger well-vascularized soft tissue for coverage of large defects after debridment of complicated groin and thigh wound as a secondary event. These modifications included either a modified laterally oriented VRAM with tongue-like skin extension or a delayed extended inferiorly based VRAM. The design of the lateral orientation is based on a well-established vascular anatomy of inferior epigastric vessels which give rise to large fasciocutaneous perforators that communicate by means of choke vessels to anterior branches of lateral intercostals vessels at a 45-degree angle to the anterior axillary line [8, 19] Also, the superior epigastric artery divides into 2-3 branches almost immediately upon entering the rectus muscle; the lateral segmental branch skirts the costal margin in the neurovascular plane and eventually becomes the eighth intercostal artery. These muscular branches give rise to musculocutaneous perforators, which also anastomose with deep inferior epigastric pedicle [20–22]. Buchel et al. have found that preservation of the origin of the rectus muscle to the pubis protected the pedicle from undo or twist [16]. The authors believe that the tongue-like skin extension design also avoids traction injury of the fibrovascular pedicle, which serves as the sole tether point after transecting the origin and insertion of the muscle. Therefore, this provides more reliability to the flap while increasing the arc of rotation. In addition, it ensured adequate perfusion to the distal portion of the flap through preservation of the continuity of the subdermal plexus supplied through periumbilical perforators from the inferior epigastric. Moreover, this extension provided coverage for the medial part of the defect. Previous studies have introduced modification on the rectus abdominis muscle flap to provide extralength, such as oblique rectus abdominis musculocutaneous (ORAM) flap [14]. Harvesting of the ORAM may be easier with less dissection and morbidity, but the authors believe that providing the distal muscular component rather than the distal thin fasciocutaneous component of the ORAM is necessary. The muscular component of the VRAM has been utilized to fill the cavity, provide adequate blood supply, and act as an antibiotic delivery system to resist infection [4, 7] in this high-risk immunocompromosed group of patients. In addition, the presence of valves in veins of the inferior epigastric system and intramuscular lymphatic bundles in the rectus muscle reduces the incidence of venous stasis, oedema, and lymphoedema in the dependant portion of these large flaps [2, 4].

The delayed phenomenon has been used for many years to enhance functional blood flow through vasodilatation of the arterial network and avoidance of complete vasoconstriction caused by catecholamine release, and hence it increases the flap reliability especially in high-risk patients [23, 24]. The delayed transverse rectus abdominis musculocutaneous (TRAM) flap has been widely described in the literature especially for breast reconstruction in high-risk patients [25]; on the other hand, standard VRAM flaps have been used for chest wall reconstruction especially for intractable radiation ulcers [26]. However, to the authors' best knowledge, there has been a lack of description for the delayed VRAM flap in literature being reported only as a case report based on the superior epigastric for chest wall reconstruction [20]. Accordingly, this would be the first report about an inferiorly based delayed extended VRAM flap which has been used in two patients harvesting an additional fasciocutaneous component from the chest wall in continuity with fasciocutaneous component of VRAM. The standard technique for harvesting the VRAM may lead to ischemia and venous congestion in the distal portion with a higher incidence of flap failure [16]. Therefore, performing the two-stage complete circumferential surgical and vascular delayed technique has provided an additional safe, reliable, and predictable extralength in this high-risk group without jeopardizing the wound healing and with no further delay in the postoperative chemotherapy treatment. The pinch technique described by Taylor et al. [8] have shown to avoid any donor site morbidity with excision of the nonperfused skin demarcated from the augmented perfused skin. The use of the delayed pre-expanded tissue has not been considered in these patients due to the marked delay in coverage and postoperative chemotherapy treatment.

The authors believe that these modifications will provide reliable extralength of soft tissue, which is tension free with a wider arc of rotation, better filling of dead space with well-vascularized tissue so as to resist infection, and stable coverage with good functional outcome.

Other modalities described for management of complicated infected wounds as skin grafting or vacuum-assisted closure would not be suitable due to the presence of dead space and exposed hardware prosthesis after debridement [2, 4, 27]. The local effects of infections and/or surgical trauma create a dearth of local soft tissue available to provide stable coverage of these complex wounds. In previous studies, trials have reported to cover proximal lower limb defects with either single or combined large lower extremities flaps [28, 29]. The local muscle flaps in this region are often hypovascular and fibrotic rendering them insufficient to provide sufficient soft tissue coverage, in addition, the use of local muscle flaps may result in loss of extremity and joint stability [7, 18, 27]. The authors believe that the use of these muscles may further compromise an already weakened extremity, making distant flaps as rectus abdominus muscle a better choice for reconstruction.

The use of free-tissue transfer is rare in these clinical scenarios; on the other, hand-free osteocutaneous fibular flaps were performed for primary skeletal reconstruction after tumor extipiration of sarcoma of the lower extremities [30] rather than soft tissue reconstruction for complicated groin wounds as a secondary event. While microvascular options could be considered, the limited availability of recipient vessels with higher incidence of vascular complications, proximity of anastomosis to potentially infected zone following previous radical surgery, opening new dissection plane, and lengthy operative time in immunocompromosed patients would preclude the use of this option in these settings as the first option [2, 4, 27]. In addition, venous drainage and tissue oedema may also be more problematic with such flaps in dependant portion of the lower extremity [2].

## 6. CONCLUSION

In conclusion, the modified VRAM flaps would offer reliable reconstructive tools for coverage of complicated primary wound with necrosis and breakdown after radical resection of pelvic and proximal lower limb musculoskeletal sarcoma. They offer larger well-vascularized soft tissue with acceptable donor site, more durable coverage with no delay in the postoperative adjuvant therapy, good functional outcome, together with overall improvement in the survival.

## Figures and Tables

**Figure 1:**
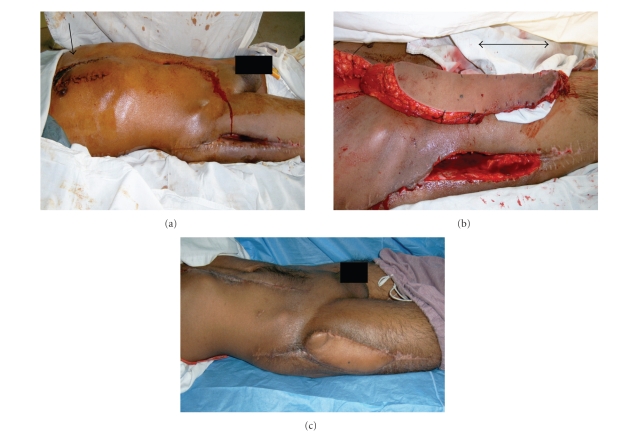
(a) A two-week picture after delay procedure (surgical and vascular delay) for extended inferiorly based VRAM flap. The arrow points to line of demarcation between nonperfused and augmented perfused skin in the parasternal fasciocutaneous extension of the VRAM flap. This extension provided additional predictable length of 7 cm for coverage of exposed plate femur after osteosarcoma resection of the proximal femur. (b) Intraoperative picture after harvesting of VRAM flap and debridement of necrotic thigh flap. The VRAM extension marked by the suture markings of the delay procedure provides the extra length avoiding tension and providing durable coverage. (c) Postoperative picture 3 months during chemotherapy course showing complete survival of the flap providing stable coverage and complete healing of the donor site.

**Figure 2:**
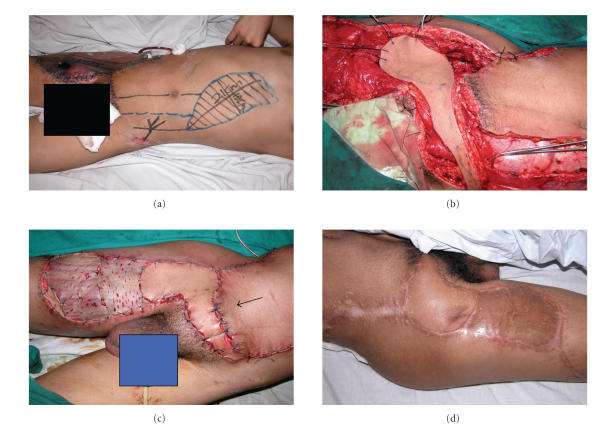
(a) Preoperative picture showing the dimensions and design of contralateral, laterally oriented VRAM with a tongue-like extension. The arrow points to the necrotic thigh flap advocated achieving adequate oncological resection for pelvic osteosarcoma. (b) Intraoperative picture showing insetting of flap after debridment of the necrotic area. The distal muscular component of the flap fills the composite defect which helps resist infection in this potentially infected area. (c) Intraoperative picture showing coverage of the defect with application of a split thickness graft on the remaining muscular bed. The arrow points to the tongue-like skin extension which avoids tension on the vascular pedicle especially after dividing the origin and insertion and ensures adequate perfusion to the distal portion of the flap. (d) Late postoperative picture 6 months after completion of chemotherapy course showing complete survival of the flap with long-term durability and complete take of the graft.
